# Emergence of ST383 carbapenem-resistant *Klebsiella pneumoniae* harbouring *bla*_NDM-5_ and *bla*_OXA-48_ carbapenemase genes in a Romanian tertiary-care hospital

**DOI:** 10.1093/jacamr/dlag079

**Published:** 2026-05-19

**Authors:** Valeriu Gheorghiță, Ana Dobrin, Violeta Melinte, Maria-Adelina Radu, Maria Cristina Văcăroiu, Irina-Florentina Talpoși, Tiberiu-Sebastian Holban, Anamaria Dobrotă, Mădălina-Maria Muntean, Brîndușa Elena Lixandru, Mircea Ioan Popa, Andrei-Alexandru Muntean

**Affiliations:** Department of Infectious Diseases IV, ‘Carol Davila’ University of Medicine and Pharmacy, Bucharest, Romania; Infectious Diseases Department, ‘Agrippa Ionescu’ Clinical Emergency Hospital, Bucharest, Romania; Department of Infectious Diseases IV, ‘Carol Davila’ University of Medicine and Pharmacy, Bucharest, Romania; Infectious Diseases Department, ‘Agrippa Ionescu’ Clinical Emergency Hospital, Bucharest, Romania; Department of Infectious Diseases IV, ‘Carol Davila’ University of Medicine and Pharmacy, Bucharest, Romania; Infectious Diseases Department, ‘Agrippa Ionescu’ Clinical Emergency Hospital, Bucharest, Romania; Department of Infectious Diseases IV, ‘Carol Davila’ University of Medicine and Pharmacy, Bucharest, Romania; Infectious Diseases Department, ‘Agrippa Ionescu’ Clinical Emergency Hospital, Bucharest, Romania; Infectious Diseases Department, ‘Agrippa Ionescu’ Clinical Emergency Hospital, Bucharest, Romania; Department of Infectious Diseases IV, ‘Carol Davila’ University of Medicine and Pharmacy, Bucharest, Romania; Infectious Diseases Department, ‘Agrippa Ionescu’ Clinical Emergency Hospital, Bucharest, Romania; Microbiology Department, ‘Agrippa Ionescu’ Clinical Emergency Hospital, Bucharest, Romania; Epidemiology Department, ‘Agrippa Ionescu’ Clinical Emergency Hospital, Bucharest, Romania; Department of Parasitology, ‘Carol Davila’ University of Medicine and Pharmacy, Bucharest, Romania; Reference Laboratory for Nosocomial and Antibiotic Resistant Infections, Cantacuzino National Military Medical Institute for Research and Development, Bucharest, Romania; Department Microbiology II-Cantacuzino NMMIRD, ‘Carol Davila’ University of Medicine and Pharmacy, Bucharest, Romania; Reference Laboratory for Enteric Bacterial Infections, Cantacuzino National Military Medical Institute for Research and Development, Bucharest, Romania; Department of Parasitology, ‘Carol Davila’ University of Medicine and Pharmacy, Bucharest, Romania; Department Microbiology II-Cantacuzino NMMIRD, ‘Carol Davila’ University of Medicine and Pharmacy, Bucharest, Romania

## Abstract

**Background:**

*Klebsiella pneumoniae* is a major cause of healthcare-associated infections in Romania, where carbapenem resistance rates exceed 63%. The dissemination of clones co-producing multiple carbapenemases poses a critical challenge to infection control and antimicrobial management.

**Objectives:**

To describe the molecular characteristics of carbapenem-resistant *K. pneumoniae* (CR*Kp*) isolates and to assess their epidemiological relevance in a Romanian tertiary-care hospital.

**Methods:**

Non-duplicate CR*Kp* isolates collected between July 2023 and May 2025 were included. Identification and antimicrobial susceptibility testing were performed according to EUCAST 2025 criteria. Isolates with reduced carbapenem susceptibility underwent WGS using Oxford Nanopore technology. MLST, resistome, virulome and plasmid profiling were analysed.

**Results:**

A total of 101 CR*Kp* isolates were included. In 55.4% (*n* = 56) of strains the resistance genotype displayed both *bla*_NDM-5_ and *bla*_OXA-48-like_ genes. WGS identified ST383 as the dominant sequence type (*n* = 31; 30.7%), with 93.5% (29/31) of ST383 clones co-harbouring *bla*_NDM-5_ and *bla*_OXA-48_ along with *bla*_CTX-M-15_ genes, primarily located on mosaic resistance–virulence plasmids, with evidence of chromosomal integration. Cefiderocol susceptibility was 90.3% by broth microdilution. All tested ST383 isolates remained susceptible to aztreonam–avibactam.

**Conclusions:**

ST383 emerged as the dominant carbapenemase-producing *K. pneumoniae* lineage and represents the first documented identification in Romania of strains co-harbouring *bla*_NDM-5_ and *bla*_OXA-48_, highlighting the emergence of a high-risk clone with substantial dissemination potential.

## Introduction

*Klebsiella pneumoniae* is one of the most frequent causes of healthcare-associated infections (HAI) in Romania, with carbapenem resistance exceeding 63%.^1,2^ This situation is likely attributable to multiple factors, including high levels of antibiotic consumption.^3^ Infections caused by carbapenem-resistant Enterobacterales are linked to substantially higher mortality, severe therapeutic limitations, prolonged hospitalization, and increased healthcare costs compared with carbapenem-susceptible strains.^4^ Over the past three years, several reports from Romania have documented a marked increase in strains co-producing NDM and OXA-48-like carbapenemases, suggesting possible clonal spread and highlighting the need for enhanced WGS–based surveillance.^5–7^ Genomic surveillance across Europe indicates that the spread of carbapenem-resistant *K. pneumoniae* (CR*Kp*) is largely driven by the dissemination of high-risk lineages, including ST11, ST15, ST101, ST258/512, and more recently ST147 and ST307, with an additional public-health threat posed by the convergence of carbapenem resistance and virulence mediated by mosaic plasmids carrying both carbapenemase and virulence genes.^8^ Between 2016 and 2018, genomic surveillance identified high-risk *K. pneumoniae* clones in Romania, including ST101 and ST15, alongside major European lineages such as ST258/512, ST11 and ST307.^9^ We report the first identification in Romania of ST383 CR*Kp* clones carrying *bla*_NDM-5_ and *bla*_OXA-48_ carbapenemase genes. This study describes the molecular characteristics and explores the potential epidemiological significance of ST383 clones detected in a tertiary-care hospital between July 2023 and May 2025.

## Materials and methods

We analysed non-duplicate CR*Kp* isolates recovered from different clinical specimens at the ‘Agrippa Ionescu’ Clinical Emergency Hospital during two study periods: July 2023–January 2024 and April 2024–May 2025.

Initial species identification and antimicrobial susceptibility testing, including meropenem (MEM) MIC determination, were performed using the VITEK 2 XL system (bioMérieux) with GN, AST-N426 and AST-N439 cards, according to the manufacturer's instructions. The antimicrobial panels included representatives of major antibiotic classes, including β-lactams (carbapenems and ceftazidime/avibactam), fluoroquinolones, aminoglycosides and polymyxins. MEM MIC values and all other susceptibility results were interpreted according to EUCAST 2025 clinical breakpoints. Isolates exhibiting decreased susceptibility to at least one carbapenem were preserved at −80°C and referred to the National Reference Centre for Nosocomial and Antibiotic-Resistant Infections at the Cantacuzino National Military Medical Institute for Research and Development (Cantacuzino NMMIRD).^10^ Prior to further analyses, strains were revitalized on Columbia Blood Agar, prepared from Oxoid dehydrated powder and supplemented with 5% sheep blood.

Species identification was performed using the Bruker Daltonics sirius Matrix-Assisted Laser Desorption Ionization-Time of Flight Mass Spectrometry (MALDI-ToF-MS) system, with IVD identification (MBT IVD Library Revision J, 2022). An overnight culture of *K. pneumoniae* was used for DNA extraction using the Monarch gDNA Spin kit T3010L (New England Biolabs). WGS was carried out using Oxford Nanopore Technology, a run using the MinION device and R10.4 flowcell and a second run using the PromethION device and R10.4 flowcell, using the 24 and 96 Rapid Barcoding Kit v14, as per manufacturer's recommendations. Bioinformatic analysis was done using a Autocycler-based assembly, and focused on MLST detection, resistome and virulome characterization and plasmid profiling.^11–16^ Reference-independent parsimony trees were generated using kSNP4.1, together with genomic data derived from NCBI AMRFinder, ResFinder, and Kleborate. The results were compiled into a database and plotted using a custom Python script.

Cefiderocol (FDC) susceptibility for all ST383 isolates (*n* = 31) was determined by disk diffusion on Mueller–Hinton E agar (bioMérieux) with 30 µg FDC disks from Oxoid Ltd. (Basingstoke, UK) and broth microdilution, using Bruker UMIC^®^ test. Susceptibility testing for aztreonam-avibactam (ATM-AVI) was performed using gradient diffusion (MIC Test Strip, Liofilchem) on Mueller–Hinton agar (Oxoid).

## Results

**Figure 1.**
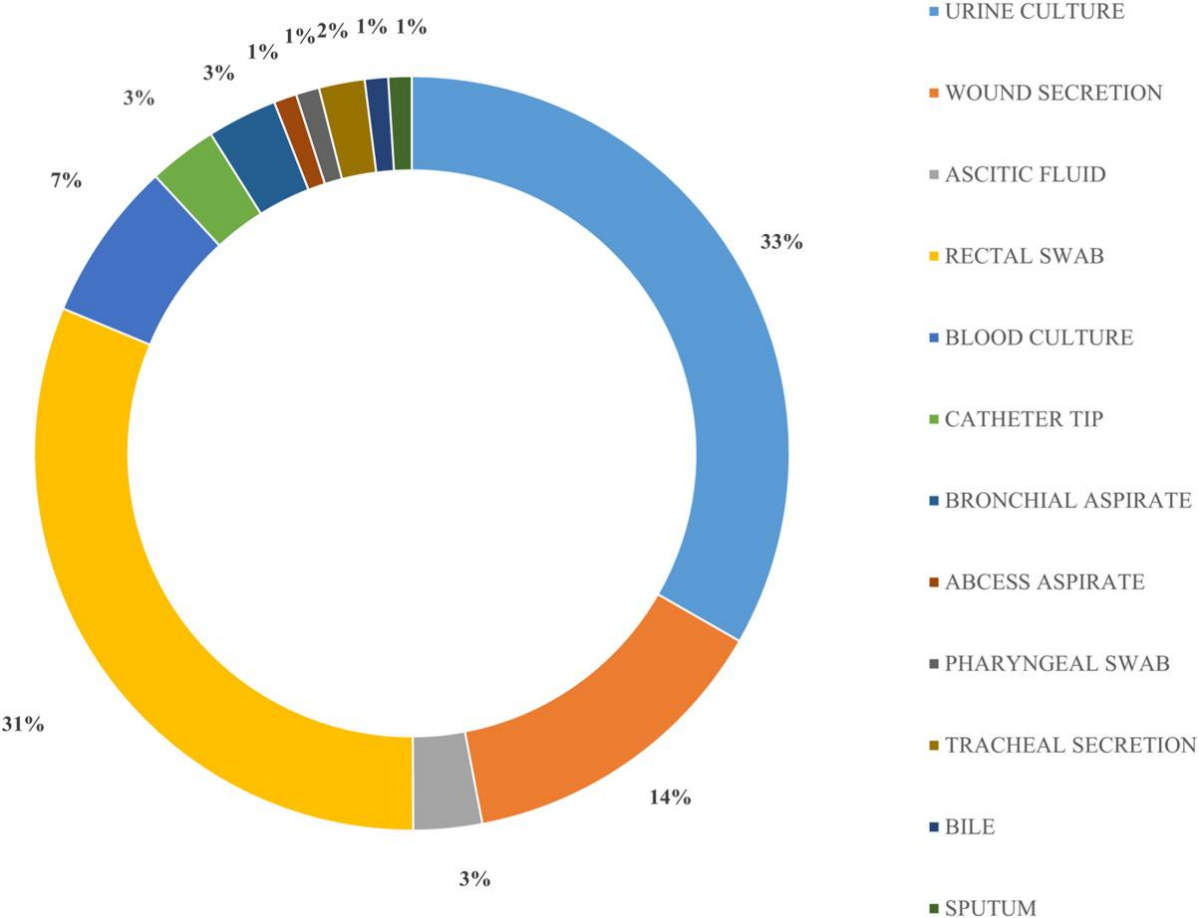
Distribution of clinical specimen sources of carbapenem-resistant *K. pneumoniae* isolates.

During the study period, 101 isolates with reduced susceptibility to at least one carbapenem were recovered from a variety of clinical specimens, most commonly urine cultures (33%) and rectal swabs (31%). Other sources included wound secretions (14%), blood cultures (7%), ascitic fluid (3%), catheter tips (3%), and bronchial aspirates (3%), while tracheal secretions (2%), bile, sputum, abscess aspirates, and pharyngeal swabs (each 1%) were less frequently represented (Figure 1).

The predominant resistance genotype consisted of the co-presence *bla*_NDM_ and *bla*_OXA-48-like_ genes (*n* = 56, 55.4%), followed by isolates carrying *bla*_OXA-48-like_ in combination with ESBL genes (*n* = 22, 21.8%) and isolates co-harbouring *bla*_NDM_ and ESBL genes (*n* = 13, 12.9%). Isolates carrying *bla*_KPC-2_, either alone (*n* = 4, 4%) or in combination with *bla*_NDM-1_ (*n* = 2, 2%), accounted for a minority of cases.

**Figure 2.**
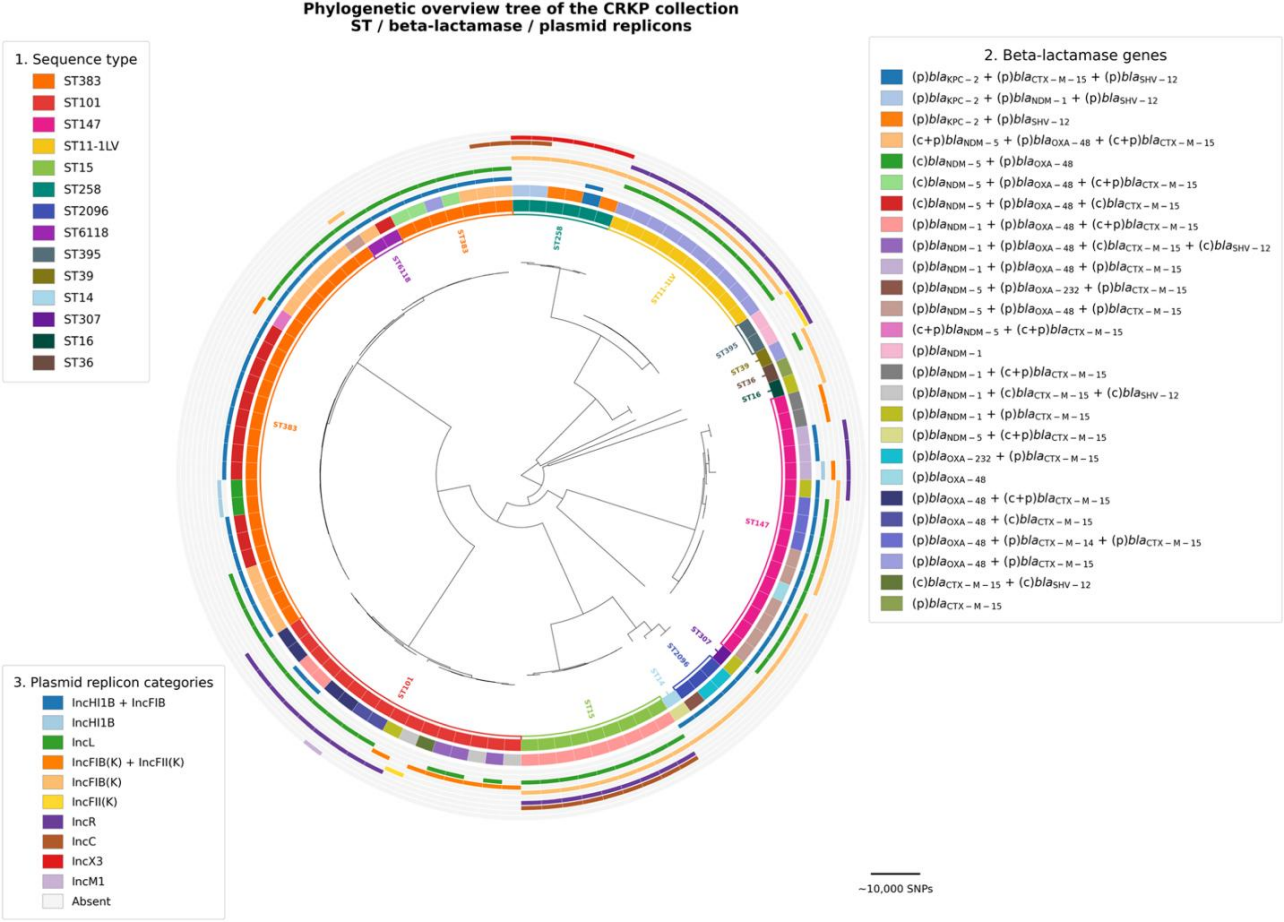
The phylogenetic overview tree of the CR*Kp* collection including sequence type, β-lactamase profile, and plasmid replicon content.

WGS identified ST383 as the predominant sequence type, accounting for 31 of 101 CR*Kp* isolates (30.7%). Notably, 29 of the 31 ST383 isolates (93.5%) carried *bla*_NDM-5_ and *bla*_OXA-48_, indicating a strong association between this lineage and the dual-carbapenemase genotype. These ST383 isolates represented 51.8% (29/56) of all CR*Kp* isolates harbouring both *bla*_NDM-5_ and *bla*_OXA-48_ genes. The same combination of carbapenemase genes was identified among ST6118 (*n* = 2; 2%), a single-locus variant of ST383, and in eight ST147 isolates (8/16; 50.0%), indicating a strong association between these sequence types and the presence of *bla*_NDM-5_ and *bla*_OXA-48_. Within ST147, additional resistance gene combinations were observed, including isolates carrying *bla*_NDM-1_ plus *bla*_OXA-48_, *bla*_NDM-1_ with ESBL genes, or *bla*_OXA-48_ and ESBL-associated genes. ST101 isolates (*n* = 17; 16.8%) also exhibited a diverse range of carbapenemase gene profiles, including *bla*_OXA-48_, or the co-occurrence of *bla*_NDM-1_ with *bla*_OXA-48_ profile. In contrast, a novel single-locus variant of ST11 (ST11-LV) (*n* = 10; 9.9%) consistently carried *bla*_OXA-48_, while ST2096 (*n* = 3; 3%) was associated with *bla*_OXA-232_, with one isolate carrying *bla*_NDM-5_. Other lineages showed more restricted resistance profiles: ST258 (*n* = 6; 5.9%) carried *bla*_KPC-2_, with a subset also harbouring *bla*_NDM-5_, whereas ST15 (*n* = 9; 8.9%) carried *bla*_NDM-1_ together with *bla*_OXA-48_. ST307 (*n* = 1; 1%) and ST395 (*n* = 2; 2%) carried *bla*_NDM-1_ alone. *bla*_CTX-M-15_ was the predominant accompanying ESBL across most sequence types, except for ST258, which carried *bla*_SHV-12_, a subset of ST101 isolates harboured both *bla*_SHV-12_ and *bla*_CTX-M-15_, while three ST147 isolates carried both *bla*_CTX-M-15_ and *bla*_CTX-M-14b_. Plasmid replicon typing revealed heterogeneous incompatibility group distributions across sequence types, with IncF-family replicons predominating among major lineages (Table 1). A phylogenetic overview tree of the CR*Kp* collection including sequence type, β-lactamase profile, and plasmid replicon content, is presented in Figure 2.

**Table 1.** Whole-genome sequencing characteristics of carbapenem-resistant *Klebsiella pneumoniae* isolates, by sequence type, resistance gene profile, and plasmid replicon content

Sequence type	*n*	Carbapenemase genes	*n*	Additional ESBL	Inc replicons identified
ST11-LV	10	*bla*_OXA-48_	10	*bla*_CTX-M-15_	IncFIB(K) IncL ColRNAI ColpVC IncR
ST14	1	*bla*_NDM-5_	1	*bla*_CTX-M-15_	IncFIB(pNDM-Mar), IncHI1B(pNDM-MAR) IncFIB(K)
ST15	9	*bla*_NDM-1_ + *bla*_OXA-48_	9	*bla*_CTX-M-15_	IncC IncFIA(HI1), repB, IncR IncL Col440II IncFIB(pKPHS1)
ST16	1	*bla*_NDM-1_	1	*bla*_CTX-M-15_	IncFIB(K) Col440II
ST36	1	—	1	*bla*_CTX-M-15_	IncFIB(K)(pCAV1099-114), RepB
ST39	1	*bla*_OXA-48_	1	*bla*_CTX-M-15_	IncFIB(K)(pCAV1099-114), RepB IncL
ST101	17	*bla*_NDM-1_	4	*bla*_CTX-M-15_	IncFIA(HI1) IncL IncFIB(pNDM-Mar), IncHI1B(pNDM-MAR) Col440II IncR, repB(R1701), IncFII(Yp) IncFIB(K), IncFII(K)
		*bla*_NDM-1_	3	*bla*_CTX-M-15_ + *bla*_SHV-12_	
		*bla*_OXA-48_	5	*bla*_CTX-M-15_	
		*bla*_NDM-1_ + *bla*_OXA-48_	2	*bla*_CTX-M-15_	
		*bla*_NDM-1_ + *bla*_OXA-48_	3	*bla*_CTX-M-15_+ *bla*_SHV-12_	
ST147	16	*bla*_NDM-5_ + *bla*_OXA-48_	8	*bla*_CTX-M-15_	IncFIB(pQil) IncFIB(pNDM-Mar), IncHI1B(pNDM-MAR) IncL IncFII(Yp) IncFIB(K)(pCAV1099-114) IncR IncFII(K)
		*bla*_NDM-1_ + *bla*_OXA-48_	2	*bla*_CTX-M-15_	
		*bla*_NDM-1_	3	*bla*_CTX-M-15_	
		*bla*_OXA-48_	3	*bla*_CTX-M-15_ + *bla*_CTX-M-14b_	
ST258	6	*bla*_KPC-2_	3	*bla*_SHV-12_	IncFIB(pQil) IncFIB(K) ColRNAI IncX3 IncC Col440II ColRNAI IncC
		*bla*_KPC-2_	1	*bla*_SHV-12_ + *bla*_CTX-M-15_	
		*bla*_KPC-2_ + *bla*_NDM-1_	2	*bla*_SHV-12_	
ST307	1	*bla*_NDM-1_	1	*bla*_CTX-M-15_	IncFIB(K) IncFIB(pNDM-Mar), IncHI1B(pNDM-MAR) IncFIB(pQil)
ST383	31	*bla*_NDM-5_ + *bla*_OXA-48_	27	*bla*_CTX-M-15_	IncFIB(pNDM-Mar), IncHI1B(pNDM-MAR) IncL IncFIB(K), IncFII(K) IncC IncFIB(K) Col440II
		*bla*_NDM-5_ + *bla*_OXA-48_	2	—	
		*bla*_OXA-48_	1	*bla*_CTX-M-15_	
		*bla*_NDM-5_	1	*bla*_CTX-M-15_	
ST395	2	*bla*_NDM-1_	2	—	IncFII(Yp) Col(CriePir75) IncR
ST2096	3	*bla*_OXA-232_	2	*bla*_CTX-M-15_	IncFIB(pNDM-Mar), IncHI1B(pNDM-MAR) IncFIB(K) ColKP3
		*bla*_NDM-5_ + *bla*_OXA-232_	1	*bla*_CTX-M-15_	
ST6118	2	*bla*_NDM-5_ + *bla*_OXA-48_	2	*bla*_CTX-M-15_	IncFIB(pNDM-Mar), IncHI1B(pNDM-MAR) IncL

Antimicrobial susceptibility testing of ST383 isolates demonstrated uniformly elevated MEM MIC values, with all isolates showing MICs ≥16 mg/L by the VITEK 2 system, consistent with phenotypic carbapenem resistance. All ST383 strains were resistant to aminoglycosides and fluoroquinolones. Colistin susceptibility was assessed only by VITEK, without confirmation by broth microdilution; therefore, an exact resistance rate could not be reliably estimated for the present cohort. However, local unpublished data from our institution suggest that approximately 80% of CR*Kp* isolates are resistant to colistin, further supporting caution when considering colistin as a reliable therapeutic option. FDC susceptibility testing revealed notable discrepancies between methodologies. By disk diffusion, 10/31 isolates were categorized as resistant, 3 fell within the area of technical uncertainty (ATU), and 18 were classified as susceptible (58.1%). In contrast, broth microdilution (BMD), the EUCAST reference method, identified only three resistant isolates (MIC range, 4–8 mg/L), whereas 28/31 isolates (90.3%) were categorized as susceptible (MIC range, 0.125–2 mg/L). Regarding the susceptibility to ATM-AVI, all tested ST383 isolates were susceptible, with MICs ranging from 0.125 to 0.5 mg/L. ST383 showed a predominant rough colony phenotype, displayed a Kleborate virulence score of 3/5 and a resistance score of 3/3.

**Figure 3.**
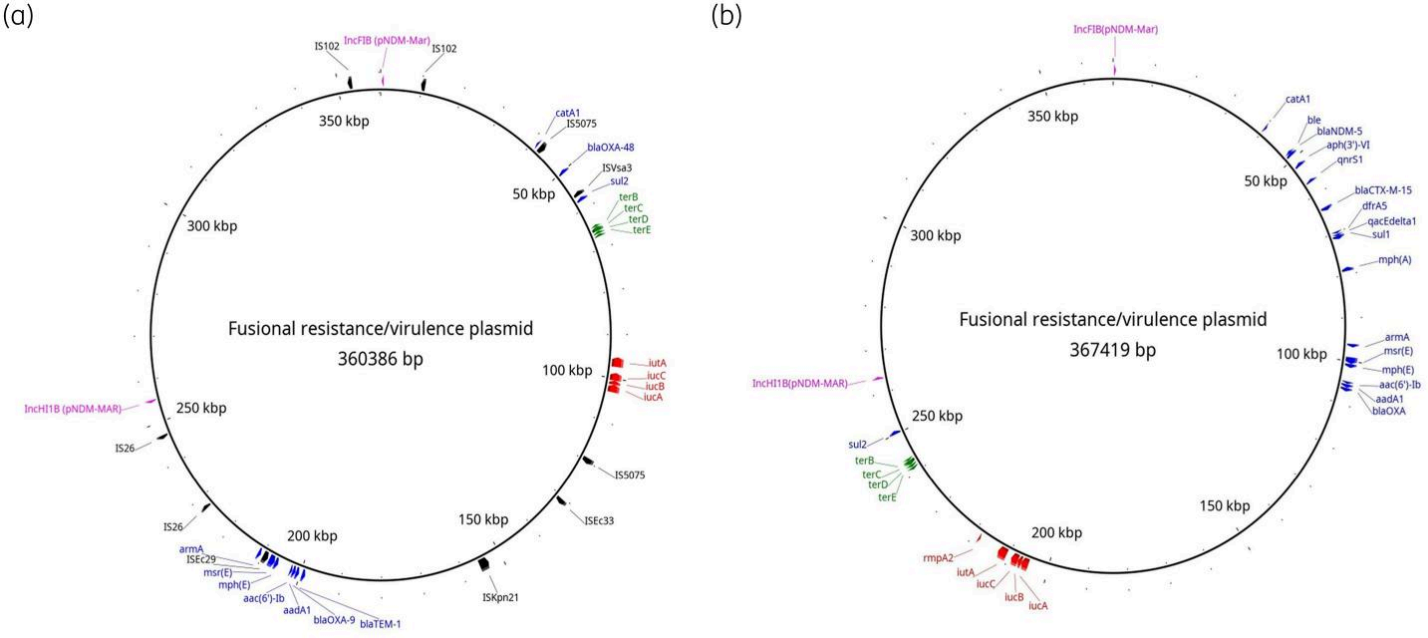
Fusional resistance and virulence plasmids in *Klebsiella pneumoniae* ST383 strains, carrying either *bla*_OXA-48_ (A) or *bla*_NDM-5_ (B) and *bla*_CTX-M-15_, along with associated virulence determinants.

Genomic analysis confirmed the presence of *bla*_NDM-5_, *bla*_OXA-48_, along with *bla*_CTX-M-15_ in most isolates (27/31), with several isolates carrying chromosomal insertions of *bla*_NDM-5_ and *bla*_CTX-M-15_ as well as carriage of these resistance genes on an IncFIB–IncHI1B plasmid (Figure 3). The chromosomal insertion containing *bla*_NDM-5_, *bla*_CTX-M-15_ is flanked by IS110 family IS4321 transposases within a region enriched in mobile genetic elements.

## Discussion

This study provides the first documented evidence of CR*Kp* ST383 clones in Romania carrying both *bla*_NDM-5_ and *bla*_OXA-48_. The localization of *bla*_NDM-5_ and *bla*_OXA-48_ on mosaic resistance–virulence plasmids, together with chromosomal integration events, indicates a lineage with enhanced potential for stable transmission and long-term persistence in healthcare settings. Such genetic configurations are increasingly recognized as key drivers of clonal success, facilitating both horizontal gene transfer and vertical inheritance of resistance determinants.

Since the first description of *K. pneumoniae* ST383 in a case report from Greece in 2010, it has progressively emerged as a multidrug-resistant lineage with significant adaptive potential. Papagiannitsis *et al*. first reported ST383 sequence co-producing VIM-4, KPC-2 and CMY-4 β-lactamases, already demonstrating its capacity to accumulate multiple resistance determinants within a single genetic background.^17^ The accumulation of resistance traits in newly emerged lineages highlights the mobility of resistance genes, especially in healthcare settings lacking effective infection control measures.^17^ A decade later, Scaltriti *et al*. described in Italy a hypermucoviscous ST383 isolate co-harbouring *bla*_NDM-5_ and *bla*_OXA-48_ marking a shift towards dual carbapenemase carriage within this lineage.^18^ Subsequent genomic studies from China identified ST383 among CR*Kp* populations, supporting its broader geographic dissemination and genomic diversification.^19^ More recent data from Qatar demonstrated the emergence and interhospital spread of ST383 strains, reinforcing its classification as a potentially high-risk lineage capable of sustained transmission in healthcare settings.^20^ Similarly, reports from Egypt have linked high-risk carbapenem-resistant clones, including ST383, with hypervirulence determinants, further expanding concerns regarding its pathogenic potential.^21^ The most compelling recent evidence comes from Central Italy, where D’Achille *et al*. identified ST383 as the exclusive lineage among sequenced isolates co-harbouring *bla*_NDM-5_ and *bla*_OXA-48_ across three different hospitals.^22^ This finding strongly supports successful clonal dissemination. Notably, the Italian cohort exhibited high FDC resistance rates (73.6%), while ATM-AVI susceptibility remained largely preserved, underscoring the therapeutic challenges posed by this clone. In contrast, ST383 isolates in our cohort demonstrated markedly higher FDC susceptibility (90.3%). This discrepancy may reflect differences in the underlying resistome composition and genetic context. Reduced FDC susceptibility has been associated with metallo-β-lactamase (MBL) production, particularly NDM variants, and may be further influenced by co-carriage of additional β-lactamases or permeability alterations.^23–25^ The absence of *bla*_SHV-12_ and other potential cefiderocol-modifying determinants in our isolates may partly explain the preserved activity observed in our setting. Variability in plasmid backgrounds, iron-transport system mutations, or local antimicrobial selective pressure may also contribute to these inter-cohort differences.

Collectively, these observations position ST383 among the expanding group of high-risk lineages associated with the carriage of multiple carbapenemase genes.

From a clinical perspective, the emergence of ST383 carrying both *bla*_NDM-5_ and *bla*_OXA-48_ carbapenemase genes highlights increasing therapeutic challenges. The concurrent presence of multiple carbapenemase genes markedly compromises the activity of most β-lactam/β-lactamase inhibitor combinations, substantially narrowing treatment options and increasing reliance on combination regimens or last-line agents. Although preserved susceptibility to ATM–AVI represents a potential therapeutic alternative, access to this combination and real-world clinical experience remains uneven across Europe, underscoring the importance of early molecular characterization to inform timely and appropriate antimicrobial therapy.^22^ Given the clinical importance of accurate FDC susceptibility assessment, adherence to EUCAST recommendations, which recognize BMD as the reference method, is essential. In our cohort, FDC susceptibility rates differed markedly according to the testing method used, with notable discrepancies between disk diffusion and BMD. This divergence underscores the variability observed between testing approaches when assessing FDC activity in CR*Kp*. However, FDC represents a valuable therapeutic option when *in vitro* susceptibility is preserved, as observed in our cohort. The accumulation of resistance determinants, particularly in isolates carrying multiple carbapenemase genes, has been consistently associated with an increased risk of clinical failure and mortality, reinforcing the need for prompt identification and optimized management strategies.^26^

The increasing burden of MBL-producing CR*Kp* in Romania likely reflects the convergence of antibiotic selective pressure, infrastructural limitations, gaps in infection prevention and control (IPC), and constrained access to advanced molecular diagnostics. The emergence of *K. pneumoniae* ST383 carrying both *bla*_NDM-5_ and *bla*_OXA-48_ aligns with broader European evidence of expanding high-risk lineages driven by carbapenemase gene dissemination and underscores how endemic healthcare settings may act as reservoirs for sustained transmission and potential cross-border spread of antimicrobial resistance. In this context, the coexistence of resistance and virulence determinants on mosaic plasmids, together with chromosomal integration events, may further complicate outbreak containment by promoting clonal stability and persistence within hospital environments.

Recently, a mosaic resistance/virulence plasmid was described harbouring *bla*_OXA-48_.^16^ Herein, we found a similar fusional plasmid containing *bla*_OXA-48_, aerobactin-associated genes and both IncHI1b (pNDM-MAR) and IncFIB (pNDM-MAR) replicon types. In isolates carrying this mosaic plasmid (*n* = 12), *bla*_NDM-5_ and *bla*_CTX-M-15_ were exclusively chromosomally located.

Genomic-based surveillance of carbapenem-resistant Enterobacterales, as outlined in the ECDC protocol for the 2025 survey (CRE25), aims to characterize the occurrence, distribution, and population dynamics of high-risk lineages across EU/EEA countries to inform risk assessment and control policies.^27^ The present findings illustrate the critical need for enhanced WGS capacity, harmonized analytical frameworks, and timely data sharing. Given the identification of closely related ST383 clones in Southern and Central Europe, coordinated cross-border collaboration and integrated genomic surveillance are essential to monitor the spread of this emerging high-risk lineage, support targeted IPC interventions, and enable timely public-health action. While limited to a single tertiary-care centre, this study adds to accumulating European evidence that ST383 represents an emerging lineage of significant clinical and epidemiological concern, warranting further multicentre and longitudinal investigation.

## Conclusion

During the study period, multiple CR*Kp* sequence types were identified, highlighting the considerable molecular heterogeneity of CR*Kp* in our institution. Notably, ST383 emerged as the dominant and clinically most relevant lineage and constitutes, to our knowledge, the first documented identification of this high-risk clone in Romania. The emergence and apparent clonal expansion of this multidrug-resistant lineage highlight its potential for sustained dissemination within healthcare environments. These findings underscore the need for continued genomic surveillance and coordinated infection control strategies to determine whether ST383 represents a recent introduction or an evolving endemic clone.

## Data Availability

Sequencing data have been deposited in the European Nucleotide Archive (ENA) under project accession number PRJEB105915, study ERP187067.
